# A Novel Probiotic *Limosilactobacillus fermentum* IOB802 and Its Postbiotic Alleviate Cognitive Impairment Induced by Scopolamine in Mice

**DOI:** 10.3390/foods14234037

**Published:** 2025-11-25

**Authors:** Yuxuan Song, Wenjing Pan, Linlin Meng, Hengyu Wu, Boyang Li, Xuemei Han, Tianmin Fu, Wu Liang, Sa Zhou, Wenjian Ma

**Affiliations:** 1 Key Laboratory of Industrial Fermentation Microbiology of the Ministry of Education, College of Biotechnology, Tianjin University of Science and Technology, Tianjin 300457, China; 12345678ovo@mail.tust.edu.cn (Y.S.); pwj238169@mail.tust.edu.cn (W.P.); mll3196@163.com (L.M.); wuhengyu971002@163.com (H.W.); 22813515@mail.tust.edu.cn (B.L.); hanxuemei2024@163.com (X.H.); 2 Tianjin Key Laboratory of Edible Probiotics, Tianjin 300301, China; futianmin@hotmail.com (T.F.); 13388059278@163.com (W.L.); 3 College of Biological and Chemical Engineering, Qilu Institute of Technology, Jinan 250200, China

**Keywords:** cognitive impairment, probiotics, postbiotics, brain–gut axis

## Abstract

Cognitive impairment is acknowledged as an early stage between normal aging and Alzheimer’s disease, emphasizing the need for prompt intervention. There is growing evidence that the gut–brain axis plays a role in regulating cognitive function, indicating that probiotics and their derivatives may impact cognitive functions through the brain–gut axis. In this study, we isolated and identified a novel bacterial strain *Limosilactobacillus fermentum* IOB802 (IOB802) from traditionally fermented pickles. This strain showed promising probiotic properties, and its postbiotic was also prepared. Both the probiotic IOB802 and its postbiotic preparation significantly improved memory and learning abilities by using a mouse model with cognitive impairment induced by scopolamine. In comparison to the scopolamine group, IOB802 and IOB802 postbiotic administration decreased acetylcholinesterase activity by 59.2% and 29.51%, increased antioxidant enzyme activity by 44.45% and 29.43%, and lowered lipid peroxidation by 44.19% and 32.53%, respectively. Moreover, IOB802 postbiotic notably boosted acetylcholine levels by 72.08%. In addition, the treatments preserved the integrity of neurons in specific regions of the hippocampus, as shown by histological analysis. The IOB802 postbiotic increased the expression of neurotrophic factors BDNF and NGF by 1.36- and 1.73-fold, while reducing the expression of inflammatory cytokines TNF-α, IL-6, and IL-1β by 2.05-, 1.85-, and 2.46-fold, respectively. Compared to the scopolamine group, IL-6 and IL-1β expression decreased by 1.32- and 2.37-fold in the IOB802 group. Additionally, IOB802, especially its postbiotic, was found to restore disrupted intestinal flora caused by scopolamine. These findings suggest that IOB802 and its postbiotic can improve cognitive function through enhancing cholinergic activity, reducing oxidative stress, providing neuroprotection, and restoring gut microbiota composition. Postbiotics, in particular, may represent a promising alternative to live probiotics for supporting cognitive health.

## 1. Introduction

Cognitive impairment encompasses deviations in higher cognitive functions including learning, memory, language, and executive functions. These deviations can result in significant learning and memory deficits and may manifest alongside conditions such as aphasia, apraxia, agnosia, or executive dysfunction [1]. The core of cognition is rooted in the proper operation of the cerebral cortex, with any elements leading to irregularities in its function and composition having the potential to induce cognitive deficits [2]. The complexity of brain function often leads to interrelated cognitive impairments, where deficiencies in one cognitive aspect can trigger abnormalities in other areas. For instance, individuals with impairments in attention and memory may also struggle with problem-solving [3,4]. Cognitive impairment presents a significant challenge in the diagnosis and management of brain diseases, arising from various factors including organic diseases and mental disorders like neurasthenia, senile dementia, and schizophrenia [5,6]. Recent research has revealed a strong correlation between gut microbiota and brain function, suggesting that dysbiosis in gut microbiota could be a pivotal factor contributing to cognitive impairment [7]. This discovery offers novel perspectives and potential avenues for treating cognitive decline through the use of probiotics.

Probiotics are beneficial microorganisms, whereas postbiotics are the metabolites of probiotics acquired through extensive processing [8]. Common types of probiotics include *lactic acid bacteria*, *lactobacillus*, *bifidobacterium*, and *enterococcus* microorganisms. The gut–brain axis denotes the bidirectional communication between the gut and the brain [9,10]. Studies suggest a close link between the gut and the central nervous system mediated by gut microbiota, influencing brain circuits and neurophysiological functions such as cognition, emotions, and executive functions. Numerous studies have highlighted the regulatory role of probiotics in neurological disorders like depression, autism, and Alzheimer’s disease (AD) [11].

Recent research has consistently documented alterations in the microbial profile of individuals suffering from depression [12]. These changes in the gut microbiota are characterized by a decline in butyrate-producing bacteria, known for their anti-inflammatory properties, alongside an elevation in pro-inflammatory markers [13]. The abundance of butyrate-producing bacteria, like *Faecalibacterium* and *Coprococcus*, can be indicative of a better quality of life [14]. Research reveals that rodents receiving fecal microbiota transplantation (FMT) from healthy donors exhibit lower inflammation levels and less severe depressive behaviors than those receiving FMT from depressed patients [15]. These findings imply that the gut microbiota’s composition may have a role in alleviating depressive symptoms by influencing factors along the brain–gut axis.

Elmira Akbari et al. conducted a study showing that continuous supplementation of probiotic milk containing *Lactobacillus acidophilus*, *Lactobacillus casei*, *Bifidobacterium bifidum*, and *Lactobacillus fermentum* for 12 weeks significantly improved the functional and metabolic status of Alzheimer’s patients with severe cognitive impairment [16]. *Lactobacillus fermentum JDFM216* had been shown to enhance cognitive performance in mice while modulating systemic immune responses and reshaping gut microbiota composition [17]. Probiotics had also demonstrated the capacity to enhance cognitive functions in both healthy individuals and those afflicted with conditions such as depression and Alzheimer’s disease [18]. A study investigating the impact of fecal microbiota transplantation in rats unveiled the significant role of gut microbiota in cognitive function. The transfer of microbiota from aged rats to young rats resulted in cognitive impairment and decreased expression of brain-derived neurotrophic factor (BDNF), whereas the transplantation of microbiota from mice to aged rats enhanced cognitive function and induced changes in the hippocampal metabolome [19,20]. These studies have all underscored a close association between probiotic activity and cognitive outcomes via the gut–brain axis.

The gut–brain axis represents a bidirectional communication network connecting the gastrointestinal tract and the central nervous system through neural, endocrine, immune, and metabolic pathways. Emerging evidence demonstrates that intestinal barrier dysfunction and gut dysbiosis can trigger systemic inflammation and alter the production of neuroactive metabolites, including short-chain fatty acids (SCFAs), neurotransmitter precursors, and tryptophan metabolites. These gut-derived signals can modulate neuroinflammation, oxidative stress, and synaptic plasticity in the hippocampus and cortex, ultimately influencing cognitive performance [21]. Conversely, cognitive disorders and stress can impact gut homeostasis through altered autonomic nervous system activity and hypothalamic–pituitary–adrenal (HPA) axis activation [22]. This reciprocal relationship positions the gut microbiota as a potential therapeutic target for cognitive enhancement and neuroprotection.

While several *Lactobacillus* species have shown cognitive benefits, *Limosilactobacillus fermentum* strains are particularly promising due to their exceptional gastrointestinal tolerance and immunomodulatory properties [23]. In this study, a novel probiotic *Lactobacillus fermentum* IOB802 was selected from traditionally fermented pickles through a systematic screening process based on multiple criteria, such as superior acid tolerance (survival at pH 2.5) and resistance to bile salts (0.3% *w*/*v*) for viability in the gut. The strain also exhibited a safe profile with no hemolytic activity and susceptibility to antibiotics. Its origin from traditional fermented foods indicates potential health benefits. Furthermore, *L. fermentum* strains are known for their production of exopolysaccharides and bioactive metabolites, which can enhance their probiotic effects [24]. The memory-enhancing effects of *Lactobacillus fermentum* IOB802 and its postbiotic were investigated in this study. The aim is to clarify the regulatory impact of IOB802 on cognitive indicators, offering a theoretical foundation for potential probiotic applications in adjunctive therapy for conditions like memory dysfunction.

## 2. Materials and Methods

### 2.1. Isolation and Identification of Strain

Fermented pickles were serially diluted with sterile physiological saline and plated on de Man–Rogosa–Sharpe (MRS) agar. After incubation at 37 °C for 24 h, colonies showing distinct morphology were picked and purified by repeated streaking. The isolates were examined by Gram staining and light microscopy for preliminary identification.

Genomic DNA was extracted using a commercial bacterial DNA extraction kit (Solarbio, Beijing, China). The 16S rRNA gene was amplified with primers 27F (5′-AGAGTTTGATCCTGGCTCAG-3′) and 1492R (5′-GGTACCTTGTACGACTT-3′). PCR reactions (25 μL) contained 17 μL ddH_2_O, 1 μL of each primer (10 μmol/L), 1 μL dNTPs (2.5 mmol/L), 0.5 μL Taq DNA polymerase (5 U/μL), 2.5 μL buffer, and 2 μL DNA template. The PCR products were purified and sequenced bidirectionally by a commercial sequencing service (China National Research Institute of Food & Fermentation Industries Corporation Limited, Beijing, China). The resulting sequences were compared with reference sequences using the NCBI BLAST+ 2.17.0 program, and phylogenetic analysis was performed with MEGA 11.0 software.

### 2.2. Acid and Bile Tolerance Test

The acid tolerance of bacterial strains was assessed following a published method with minor modifications [25]. Overnight cultures were inoculated (2%, *v*/*v*) into MRS-L broth adjusted to pH 2.0, 2.5, 3.0, or 7.0 (control). Cultures were incubated at 37 °C, and bacterial survival was monitored at 0, 2, 4, and 8 h by measuring optical density at 600 nm (OD_600_). Uninoculated broth at each pH served as the blank control. Strains with higher survival rates under acidic conditions were considered acid-tolerant. All assays were performed in triplicate, and results are expressed as mean ± standard deviation (SD).

For bile salt tolerance, acid-resistant strains were inoculated (2%, *v*/*v*) into modified MRS broth containing bile salts at 0%, 0.1%, 0.3%, or 0.5% (*w*/*v*). Cultures were incubated at 37 °C, and growth was measured at 0, 2, 4, and 8 h by OD_600_. Uninoculated broth served as the blank control. Strains maintaining growth under bile salt stress were selected for further studies. All assays were performed in triplicate, and results are expressed as mean ± SD.

### 2.3. Simulated Gastric and Intestinal Fluids Tolerance Test

Simulated gastric juice (pH~2.0, containing pepsin) and simulated intestinal juice (pH~7.5, containing bile salts and pancreatin) were prepared according to standard protocols. Acid- and bile salt-tolerant strains were activated and inoculated (2%, *v/v*) into each medium. Cultures were incubated at 37 °C, with uninoculated medium as the blank control. Bacterial survival was monitored at 0, 2, 4, and 8 h by measuring OD_600_. All assays were performed in triplicate, and results are expressed as mean ± SD.

### 2.4. Safety Assessment

After three consecutive subcultures in modified MRS broth, the strain was streaked on Columbia blood agar and incubated at 37 °C for 24 h. Hemolytic activity was examined, and the absence of α- or β-hemolysis was considered indicative of safety. Antibiotic susceptibility was evaluated using the Kirby–Bauer disk diffusion method. A 200 µL bacterial suspension (1 × 10^7^ CFU/mL) was spread evenly on MRSC agar, and antibiotic disks were applied aseptically. Plates were incubated at 37 °C for 48 h, and inhibition zone diameters were recorded. Both assays were performed in triplicate and served as preliminary safety indicators of the probiotic candidate.

### 2.5. Preparation of Probiotic Postbiotics

For postbiotic preparation, activated *Limosilactobacillus fermentum* IOB802 was inoculated at 5 × 10^7^ CFU/mL into a fluid medium and fermented for 32 h. The fermented IOB802 underwent high-temperature drying to a final moisture content of ≤10%, yielding the postbiotic powder.

### 2.6. Animals and Group Allocation

In this study, a total of 50 SPF-grade male Kunming (KM) mice (4 weeks old and weighing between 18 and 22 g) were sourced from Tianjin YiShengYuan Bio-Technology Co., Ltd. (Tianjin, China). The mice were housed in standard conditions (temperature maintained at 22 ± 1 °C, humidity kept at 55–65%, with a 12/12 h light/dark cycle), and provided with free access to food and movement. All animal experiments were performed in accordance with the Guide for the Care and approved by the Ethics Review Board for Animal Studies of the Tianjin University of Science & Technology (approval number SWXY-20231220101).

To investigate the effects of IOB802 probiotic powder and postbiotic powder on memory and cognitive function in mice, 50 mice were randomly assigned to five groups, each comprising 10 mice. The control group was fed with a standard diet gavaged with 200 μL PBS for 30 d. The model and treatment groups were, respectively, fed with a standard diet gavaged with 200 μL PBS (model group), 200 μL *Lactobacillus fermentum* IOB802 of at a concentration of 3 × 10^9^ CFU/mL, 200 μL *Lactobacillus fermentum* IOB802 postbiotic of at a concentration of 3 × 10^9^ CFU/mL, 200 μL of Piracetam at a daily dose of 100 mg/kg (treatment groups) for 30 d. The control group received saline injections, while the model and treatment groups were injected with scopolamine, followed by behavioral tests. Subsequently, brain tissue, serum, and organ tissue samples were collected from the mice for physiological index analysis.

### 2.7. Memory Assessment of KM Mice

Two complementary behavioral tests were employed to comprehensively evaluate hippocampus-dependent spatial memory (MWM) and emotional associative memory (step-down test), covering the primary cognitive domains affected by cholinergic dysfunction. The behavioral paradigms were based on previous studies [26,27].

#### 2.7.1. The Morris Water Maze (MWM) Test

The MWM test comprises the place navigation and spatial exploration tests. Prior to testing, the MWM is filled with water. The water pool is divided into four quadrants with the center as the origin. A circular platform is placed in the second quadrant. On the day following the final gavage administration, control group and the model and treatment groups were injected with saline and scopolamine, respectively. Mice are introduced into the MWM from different quadrants sequentially, and timing commences upon release. When the mouse’s limbs are entirely on the platform and the stay is over 2 s, the time spent on the platform is recorded as the escape latency. If the platform is not located within 60 s, a maximum latency of 60 s is assigned. The spatial navigation experiment is carried out for 5 days with the same method.

The day after the spatial navigation test, the spatial exploration experiment is conducted. The platform area is first outlined on a computer, and the submerged platform is removed. Mice are randomly placed into the water from one of the quadrants, with the platform located in the second quadrant. The total time and distance spent in the second quadrant within 60 s are recorded, as well as the time taken to cross the hidden platform area to evaluate the mice’s memory of the target platform. The mice’s movement trajectory within 60 s is also recorded to assess their activity trend. All data are recorded using animal behavior recording software.

#### 2.7.2. The Step-Down Test

On the second day subsequent to the final gavage, control group and the model and treatment groups were injected with saline and scopolamine, respectively. Following a 20 min interval, the mice underwent the step-down test, consisting of both a training and testing phase. Sequentially, mice from each group were placed on the copper grid within the step-down test chamber, where they were allotted 3 min for environmental adaptation. After adaptation, a 24-volt current was applied to the copper grid, causing the mice to receive an electric shock and jump onto the insulated platform. Each experiment lasts for 120 s. After a 5-day training period, the test was conducted on the 6th day in the same manner, the mice will memorize to stay on the insulated platform. During the testing phase, the number of times the mouse jumped down from the insulated platform after the current is applied is recorded as the number of errors. The time when the mouse first jumps down from the platform is recorded as the Incubation period.

### 2.8. Elisa

After completing the behavioral experiments, the mice were euthanized, and brain tissue was collected and homogenized through grinding. The levels of brain-derived neurotrophic factor (BDNF) and phosphorylated cAMP response element-binding protein (p-CREB) in the brain tissue were quantified following the manufacturer’s protocol (Bainianshengwu, Chongqing, China).

### 2.9. The Detection of Neurochemical Indicators

After euthanizing the mice, serum, brain tissue, and liver tissue were collected and homogenized through grinding. Acetylcholine (Ach) levels and acetylcholinesterase (AChE) activity in the brain tissue are quantified following the manufacturer’s protocol (Jiancheng, Nanjing, China). Moreover, serotonin (5-HT), γ-aminobutyric acid (GABA), superoxide dismutase (SOD) activity, malondialdehyde (MDA) levels in the serum, and the activities of glutamic-pyruvic transaminase (GPT) and glutamic-oxaloacetic transaminase (GOT) in the serum are assessed by using the corresponding reagent kitskit (5-HT, Ruixin, Quanzhou, China; GABA, Aidisheng, Yancheng, China; SOD and MDA, Solarbio, Beijing, China; GOT and GPT, Jiancheng, Nanjing, China).

### 2.10. HE Staining

Brain tissues were collected immediately after euthanasia of KM mice by cervical dislocation, following standard laboratory procedures. The whole brain was rapidly removed on an ice-cooled surface to minimize postmortem changes. After isolation, the brain was bisected along the mid-sagittal plane, and one hemisphere was immersed in 4% paraformaldehyde (PFA) at 4 °C for 24 h. Then, the tissue was placed in xylene for transparency, subsequently fixed, and embedded in liquid paraffin. The paraffin blocks were cuted into 5-micrometer sections, dewaxed, and stained with hematoxylin. The sections were then dehydrated with ethanol, cleared with xylene, and finally mounted to obtain the slice samples. These samples were used to assess cellular damage within the hippocampus. Quantitative analysis of these sections was performed using ImageJ 1.54p software.

### 2.11. Nissl Staining

According to the processing steps for hippocampal tissue in Section 2.10, paraffin-embedded hippocampal sections were stained with Nissl stain for 2–5 min and rinsed in running water. The sections were then differentiated in 0.1% glacial acetic acid until appropriate contrast was obtained. After removing excess liquid, slides were dried at 65 °C, mounted with neutral balsam, and examined under a light microscope. Quantitative analysis of these sections was performed using Fiji software.

### 2.12. Quantitative Real-Time Polymerase Chain Reaction (qRT-PCR) Analysis

Total RNA was extracted from mouse brain and colon tissues using TRIzol reagent (ZOMANBIO, Beijing, China) and reverse-transcribed into cDNA with a high-capacity cDNA synthesis kit (Abcam, Cambridge, UK). Quantitative real-time PCR was carried out with SYBR Green Master Mix (Mei5 Biotechnology, Beijing, China) on QuantStudio 1 Plus (Thermo Fisher, Waltham, MA, USA). Glyceraldehyde-3-phosphate dehydrogenase (GAPDH) was used as the reference gene, and relative mRNA expression was calculated using the 2^−ΔΔCt^ method. Primer sequences were synthesized by Genewiz (Suzhou, China) and were presented in Table 1.

### 2.13. Measurement of Short-Chain Fatty Acids (SCFAs)

Standard stock solutions of acetic, propionic, isobutyric, butyric, isovaleric, and valeric acids were prepared and analyzed by gas chromatography to establish calibration curves of concentration versus peak area. Fresh fecal samples (100 mg) were homogenized with 1 mL PBS and centrifuged at 10,000 rpm for 5 min. An aliquot of the supernatant was transferred to a new vial, mixed with 250 µL of 15% (*v*/*v*) H_2_SO_4_ in methanol, and derivatized. SCFAs were quantified using a Fuli gas chromatograph equipped with an RB-614 SCFA column and a flame-ionization detector. The injector was set at 200 °C with a 20:1 split ratio, and nitrogen was used as the carrier gas at a flow rate of 2 mL/min. The detector was maintained at 220 °C with hydrogen and air supplied at 30 and 300 mL/min, respectively.

### 2.14. DNA Extraction and 16S rRNA Gene Amplification

Sample processing and sequencing were carried out by Shanghai Majorbio Bio-Pharm Technology Co., Ltd. (Shanghai, China). Total microbial DNA was extracted from colonic contents using the E.Z.N.A.^®^ Soil DNA Kit (Omega Bio-Tek, Norcross, GA, USA) according to the manufacturer’s protocol. DNA quality was assessed by 1% agarose gel electrophoresis, and concentration and purity were determined with a NanoDrop 2000 spectrophotometer (Thermo Scientific, USA). The V3–V4 hypervariable region of the bacterial 16S rRNA gene was amplified with primers 338F (5′-ACTCCTACGGGAGGCAGCAG-3′) and 806R (5′-GGACTACHVGGGTWTCTAAT-3′). Amplicons were sequenced on the Illumina NextSeq 2000 platform. Raw reads were filtered and assembled using UPARSE v7.1, clustered into operational taxonomic units (OTUs) at 97% sequence identity, and taxonomically assigned with the RDP Classifier (confidence threshold = 0.7). Downstream bioinformatic analyses were performed on the Majorbio Cloud Platform.

### 2.15. Statistical Analysis

GraphPad Prism 9.0 software was utilized for data processing and graphing. Data were depicted as mean ± standard deviation (SD). Statistical significance was evaluated through one-way ANOVA and two-way ANOVA analyses, with subsequent application of Dunnett’s post hoc test. Statistical significance was denoted by * *p* < 0.05, ** *p* < 0.01, and *** *p* < 0.001, indicating varying levels of significance.

## 3. Results

### 3.1. L. fermentum IOB802 Exhibited Favorable Probiotic Properties

**Figure 1:**
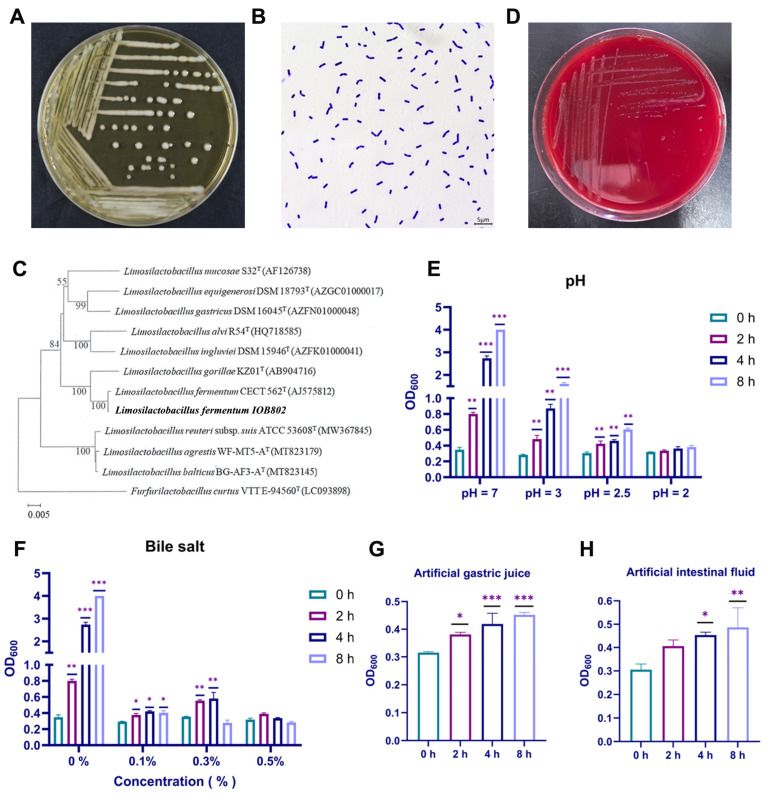
Probiotic IOB802 exhibited favorable probiotic characteristics. (**A**) The colonies of *L. fermentum* IOB802 on MRS agar. (**B**) Gram staining of *L. fermentum* IOB802. (**C**) The phylogenetic tree was constructed based on 16S rDNA sequencing result of *L. fermentum* IOB802 (This strain was indicated in bold font). (**D**) Hemolysis test of IOB802. (**E**) Acid tolerance assays of *L. fermentum* IOB802. (**F**) Bile-salt tolerance assays of *L. fermentum* IOB802. (**G**) Survival rate of IOB802 in simulated gastric fluid. (**H**) Survival rate of IOB802 in simulated intestinal fluid. Data are presented as mean ± SD. Statistical significance was assessed using one-way ANOVA followed by Dunnett’s post hoc test. * *p* < 0.05, ** *p* < 0.01, and *** *p* < 0.001 compared to the 0 h group.

We screened and isolated a novel bacterial strain from traditionally fermented pickles. The colonies of this strain were circular, opaque, milky-white, moist, convex, and had entire margins on MRS agar (Figure 1A). Gram staining revealed that the bacteria was short and blue-purple rods, consistent with Gram-positive bacteria (Figure 1B). The phylogenetic tree constructed based on 16S rRNA gene sequencing revealed that this strain had high homology with *Limosilactobacillus fermentum CECT526T* (AJ575812) and considered to belong to *Limosilactobacillus* sp. (Figure 1C). The strain was therefore identified as *L. fermentum* IOB802. We firstly evaluated the safety of *L. fermentum* IOB802 by antibiotic susceptibility and hemolytic activity analysis. The results showed that *L. fermentum* IOB802 was sensitive to ampicillin and clarithromycin, moderately sensitive to erythromycin and oxacillin, and insensitive to clindamycin, streptomycin, and fleroxacin (Table S1). No hemolytic zones were observed around colonies grown on blood agar, indicating that *L. fermentum* IOB802 did not cause hemolysis and was relatively safe (Figure 1D).

Furthermore, the gastrointestinal adaptability of *L. fermentum* IOB802 was assessed through bile-salt and acid tolerance analysis. The results showed that *L. fermentum* IOB802 grew well at bile concentrations ≤ 0.1% and pH ≥ 3.0, whereas growth was reduced at ≥0.3% bile or at pH 2.0 (Figure 1E,F). The survival properties in simulated gastric and intestinal fluids was also observed. As illustrated in Figure 1G,H, the activity of *L. fermentum* IOB802 were obviously increased after 2 h and remained stable thereafter in simulated gastric fluid and occurred a significant improvement after 4 h in simulated intestinal fluid, indicating that the strain has good acid and bile salt tolerance in the gastrointestinal tract. These results suggested that *L. fermentum* IOB802 was safe and exhibited favorable probiotic properties.

### 3.2. IOB802 and Its Postbiotics Improve Memory Performance in the Step-Down Test

The gut–brain axis is increasingly recognized as a key regulatory pathway linking intestinal homeostasis to cognitive function and neuroinflammatory processes. Among probiotic candidates, *Limosilactobacillus fermentum* have received considerable attention due to their reported immunomodulatory properties and potential neurological benefits [28,29]. Based on these research results, we investigated the functions of *L*. *fermentum* IOB802 in modulating the microbial community and improving cognition. Scopolamine, an anticholinergic drug, produces transient memory impairment by antagonizing muscarinic acetylcholine receptors, providing a model for evaluating cognitive-enhancing agents [30]. In this study, we investigated the potential preventive benefits of IOB802 and its postbiotic on the cognitive impairment model induced by scopolamine. The schematic illustration of the treatment of KM mice showed that IOB802 or its postbiotic was administered for 23 days prior to cognitive challenge, allowing establishment of gut microbiota modulation and systemic metabolic changes before scopolamine-induced injury (Figure S1).

The step-down test is a well-established paradigm for evaluating fear-associated memory in rodents. We employed this test to assess whether IOB802 and its postbiotic could mitigate the cognitive deficits induced by scopolamine administration. As shown in Table 2, scopolamine injection significantly impaired compared to the control group, as evidenced by a marked reduction in incubation period (81.52 ± 21.99 s vs. 114.61 ± 4.62 s; *p* < 0.05) and a significant increase in error frequency (2.40 ± 1.14 vs. 0.71 ± 0.49; *p* < 0.05). These results confirm the successful establishment of our cognitive impairment model. Treatment with IOB802 significantly reversed these scopolamine-induced deficits, increasing the incubation period to 105.96 ± 12.20 s (*p* < 0.05) and reducing error frequency to 0.88 ± 0.35 (*p* < 0.05). Similarly, the IOB802 postbiotic significantly improved memory performance, with an incubation period of 108.39 ± 9.80 s (*p* < 0.05) and error count of 1.00 ± 0.63 (*p* < 0.05). The positive control piracetam also exhibited significant memory-enhancing effects with comparable efficacy to both treatments. Moreover, no significant differences in body weight were observed among the groups during the study (Figure S2), suggesting that all treatments were well tolerated and did not affect the overall health of the animals.

Interestingly, IOB802 demonstrated slightly superior performance to piracetam in reducing error frequency, while the IOB802 postbiotic showed the greatest improvement in incubation period among all treatment groups. These findings suggest that both *L. fermentum IOB802* and its postbiotic possess considerable potential for ameliorating learning and memory deficits, with efficacy comparable or potentially superior to the established nootropic agent piracetam.

### 3.3. IOB802 and Its Postbiotic Enhance Spatial Learning and Memory in the Morris Water Maze

The Morris water maze (MWM) test was employed to assess the effects of IOB802 and its postbiotics on long-term memory and spatial cognition abilities, which are primarily dependent on hippocampal integrity. The test consisted of two phases: the place navigation test (conducted over 5 consecutive days) to evaluate spatial learning ability, and the spatial exploration test (conducted on day 6) to assess memory retention.

**Figure 2:**
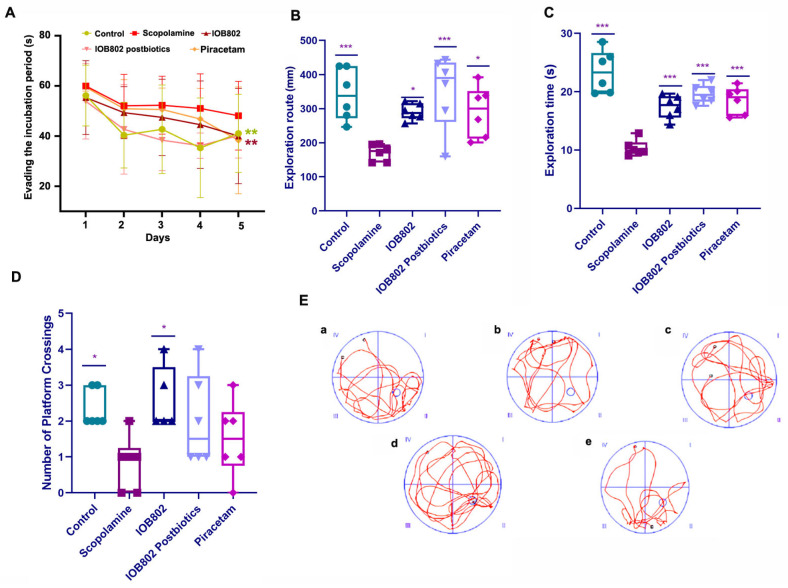
Impact of probiotic IOB802 and its postbiotics on spatial learning and memory in the Morris water maze test. (**A**) Escape latency during the 5-day acquisition phase of the Morris water maze test. Shorter escape latencies indicate better spatial learning ability. ** *p* < 0.01 compared to the Scopolamine group. (**B**) Total distance traveled in the target quadrant (where the platform was previously located) during the spatial exploration test. Compared to the Scopolamine group, * *p* < 0.05 and *** *p* < 0.001. (**C**) Time spent in the target quadrant during the spatial exploration test. Longer exploration time indicates better memory retention of the platform location. Compared to the Scopolamine group, *** *p* < 0.001. (**D**) Number of crossings over the former platform location during the spatial exploration test. More crossings indicate better spatial memory precision. Compared to the Scopolamine group, * *p* < 0.05. (**E**) Representative swimming trajectories during the spatial exploration test: (**a**) Control group, (**b**) Scopolamine group, (**c**) IOB802 group, (**d**) IOB802 postbiotic group, and (**e**) Piracetam group. The target quadrant is in the upper right corner of each panel. For (**B**–**D**), data are presented as mean ± SD (n = 6 per group). Statistical significance was assessed using one-way ANOVA, followed by Dunnett’s post hoc test.

Place navigation performance: During the 5-day training period, all groups showed a progressive decrease in escape latency, indicating learning of the platform location (Figure 2A). However, the learning curves differed significantly between groups. The scopolamine-treated group exhibited markedly impaired learning, as evidenced by consistently longer escape latencies compared to the control group. Treatment with either IOB802 or its postbiotics significantly ameliorated the scopolamine-induced deficit. By day 5, both the IOB802 group and IOB802 postbiotic group exhibited significantly shortened escape latencies in comparison to the scopolamine group. The piracetam group showed similar improvements, confirming the validity of our experimental model.

Spatial exploration performance: In the spatial exploration test, the platform was removed to assess memory retention through several parameters: time spent in the target quadrant (where the platform was previously located), distance traveled in the target quadrant, and the number of platform location crossings (Figure 2B–D). The scopolamine group showed significant memory impairment, spending less time and traveling shorter distances in the target quadrant compared to the control group. Additionally, the scopolamine group crossed the former platform location significantly fewer times, indicating compromised spatial memory. Both the IOB802 and its postbiotic significantly reversed these deficits. The IOB802 postbiotic group showed the most pronounced effect, with exploration time in the target quadrant nearly equivalent to the control group (*p* < 0.001 compared to scopolamine group). Similarly, both the IOB802 and postbiotic groups demonstrated significantly increased exploration distance and platform crossings compared to the scopolamine group.

Swimming trajectory analysis: The swimming trajectories provided further qualitative evidence of treatment efficacy (Figure 2E). Control mice exhibited focused search patterns concentrated in the target quadrant, indicating intact spatial memory. In contrast, scopolamine-treated mice displayed random swimming patterns with no apparent preference for the target quadrant, suggesting severe memory impairment. Mice treated with IOB802 and its postbiotics demonstrated more purposeful searching behavior, with swimming patterns more closely resembling those of control mice. Notably, the IOB802 postbiotic group showed the most focused search strategy, with trajectories indicating strong preference for the target quadrant, even surpassing the positive control piracetam group in search efficiency. Collectively, these results demonstrate that both *L. fermentum* IOB802 and its postbiotic effectively ameliorate scopolamine-induced deficits in spatial learning and memory, with the postbiotic treatment showing particularly robust effects on memory retention.

### 3.4. IOB802 and Its Postbiotic Normalize Neurochemical Markers Associated with Cognitive Function

**Figure 3:**
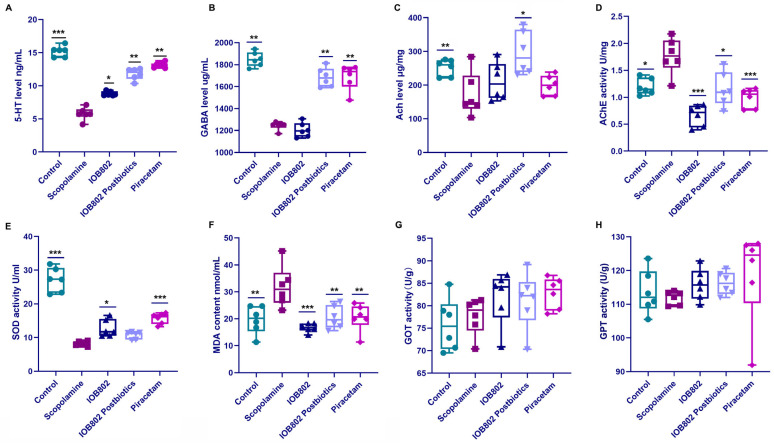
Changes in neurochemical markers of cognitive dysfunction induced by scopolamine after feeding with probiotics and postbiotics (**A**) The 5-HT content in serum. Compared to the Scopolamine group, * *p* < 0.05, ** *p* < 0.01, and *** *p* < 0.001. (**B**) The GABA content in serum. Compared to the Scopolamine group, ** *p* < 0.01. (**C**) The ACh content in brain tissue. Compared to the Scopolamine group, * *p* < 0.05 and ** *p* < 0.01. (**D**) The AchE activity in brain tissue. Compared to the Scopolamine group, * *p* < 0.05 and *** *p* < 0.001. (**E**) The SOD activity in serum. Compared to the Scopolamine group, * *p* < 0.05 and *** *p* < 0.001. (**F**) The MDA content in serum. Compared to the Scopolamine group, ** *p* < 0.01 and *** *p* < 0.001. (**G**) and (**H**) GOT and GPT activity in liver tissue, respectively. Data are presented as mean ± SD (n = 6 per group). Statistical significance was assessed using one-way ANOVA followed by Dunnett’s post hoc test.

Neurotransmitters are chemical messengers that traverse synapses to modulate mood, motor control, and memory; among them, 5-hydroxytryptamine (5-HT), γ-aminobutyric acid (GABA), and acetylcholine (ACh) are essential for proper brain function [31]. We therefore quantified serum 5-HT and GABA after each intervention. Relative to the Control group, scopolamine significantly reduced both transmitters. Compared with the scopolamine group, IOB802 postbiotic treatment restored 5-HT and GABA to normal values, whereas live IOB802 only slightly elevated the level of 5-HT (Figure 3A,B). Thus, scopolamine-induced cognitive impairment is accompanied by a down-regulation of circulating 5-HT and GABA, an effect fully reversed by IOB802 postbiotic supplementation.

The cholinergic system plays a pivotal role in learning and memory processes. As a pivotal neurotransmitter and signaling molecule in the cholinergic system, acetylcholine (ACh) regulates cognitive and emotional functions by facilitating signal transmission between neurons [32]. ACh is synthesized from acetyl-CoA and choline under the action of choline acetyltransferase and is released into the synaptic cleft [33]. Once the neurotransmission is completed, acetylcholinesterase (AChE) rapidly degrades acetylcholine (ACh) to terminate the signal transmission. Disturbances in this system are strongly associated with cognitive impairment [34]. We therefore examined whether IOB802 and its postbiotic could normalize these cholinergic markers in scopolamine-treated mice. As shown in Figure 3, scopolamine administration significantly reduced ACh levels in brain tissue compared to the control group, confirming the anticholinergic mechanism of scopolamine-induced cognitive impairment. Treatment with IOB802 postbiotic significantly reversed this reduction, while IOB802 and piracetam showed positive trends that did not reach statistical significance (Figure 3C). The AChE activity was significantly elevated in the scopolamine group compared to controls (Figure 3D), suggesting a compensatory response to cholinergic blockade. All three treatments—IOB802, postbiotics, and piracetam—significantly reduced AChE activity compared to the scopolamine group, with IOB802 demonstrating the most pronounced effect (Figure 3D).

Oxidative stress contributes to neuronal damage and cognitive decline through excessive free radical production and lipid peroxidation. Superoxide dismutase (SOD) is a crucial antioxidant enzyme that scavenges superoxide anion radicals to mitigate oxidative damages [35], while malondialdehyde (MDA) serves as a marker for lipid peroxidation and cellular damage [36]. To determine the therapeutic effects of IOB802 and its postbiotics, the SOD and MDA levels in mice blood were measured. Serum SOD activity was reduced in the scopolamine group compared to controls (Figure 3E), indicating compromised antioxidant capacity. Treatment with IOB802 restored SOD activity, with efficacy comparable to piracetam. The IOB802 postbiotics showed a positive trend that did not achieve statistical significance. MDA levels were significantly elevated in the scopolamine group compared to the controls (Figure 3F), reflecting increased oxidative damage. All three treatments (IOB802, IOB802 postbiotics, and piracetam) reduced MDA levels, with IOB802 showing superior efficacy to the standard drug piracetam. These findings demonstrate that IOB802 and its postbiotics can mitigate MDA production, thereby reducing cumulative neuronal damage and potentially ameliorating neuronal dysfunction and enhancing cognitive function (Figure 3F).

To assess potential hepatotoxicity, we measured serum levels of glutamic-oxaloacetic transaminase (GOT) and glutamic-pyruvic transaminase (GPT), which are sensitive indicators of liver function. As shown in Figure 3G,H, neither IOB802 nor its postbiotic significantly altered GOT and GPT levels compared to the control group, indicating no detectable hepatotoxicity at the administered doses.

Collectively, these results suggest that IOB802 and its postbiotic ameliorate cognitive dysfunction partly through normalizing cholinergic neurotransmission and enhancing antioxidant defense mechanisms. The differential efficacy of the treatments across various markers suggests potentially complementary mechanisms of action between IOB802 and its postbiotic derivatives.

### 3.5. IOB802 and Its Postbiotics Attenuate Hippocampal Neuronal Damage

**Figure 4:**
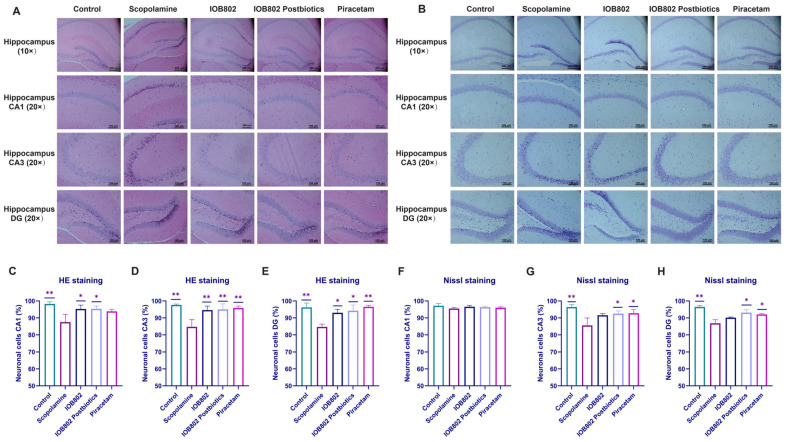
Staining of the hippocampal CA1, CA3 and DG regions in mice. (**A**) HE staining of the CA1, CA3 and DG subfields across groups to assess cell dispersion and morphology. (**B**) Nissl staining of the CA1, CA3 and DG subfields across groups to assess cell dispersion and neuronal integrity. (**C**) Number of neuronal cells in the CA1 region of the hippocampus as measured by HE staining. (**D**) Number of neuronal cells in the CA3 region of the hippocampus as measured by HE staining. (**E**) Number of neuronal cells in the DG region of the hippocampus as measured by HE staining. (**F**) Number of neuronal cells in the CA1 region of the hippocampus as measured by HE staining by Nissl staining. (**G**) Number of neuronal cells in the CA3 region of the hippocampus as measured by Nissl staining. (**H**) Number of neuronal cells in the DG region of the hippocampus as measured by Nissl staining. Data represent mean ± SD. Statistical significance was assessed using one-way ANOVA, followed by Dunnett’s post hoc test for (**C**–**H**). Compared to the Scopolamine group, * *p* < 0.05, ** *p* < 0.01.

The hippocampus is a critical brain structure for learning and memory, with distinct regions CA1, CA3 and dentate gyrus (DG) contributing differentially to cognitive functions [37]. To visualize the structural effects of scopolamine on the hippocampus, HE and Nissl staining were carried out across the CA1, CA3 and DG subregions, and the corresponding images and quantitative analyses are shown in Figure 4. As presented in Figure 4A,B, control mice displayed well-organized neuronal layers with intact soma morphology, clear nuclear structure and abundant Nissl substance. Scopolamine exposure produced marked alterations, including reduced neuronal density, soma shrinkage, irregular cell contours and loss of Nissl bodies. Treatment with IOB802 or its postbiotics alleviated these structural abnormalities. HE staining showed improved neuronal arrangement and preservation of soma shape, while Nissl staining confirmed partial recovery of nuclear integrity and cytoplasmic Nissl substance. In particular, the postbiotic group exhibited morphological features that were closest to the control condition. These observations were consistent with the quantitative results (Figure 4C–H), which demonstrated increased neuronal survival in the CA1, CA3 and DG regions following treatment. Collectively, IOB802 and its postbiotic counteract scopolamine-evoked neuronal damage and cognitive decline, with the postbiotic offering superior hippocampal neuroprotection.

### 3.6. IOB802 and Its Probiotics Affect the Expression Level of Neurotrophic Factors, Pro-Inflammatory Genes, and Intestinal Tight Junction Component

**Figure 5:**
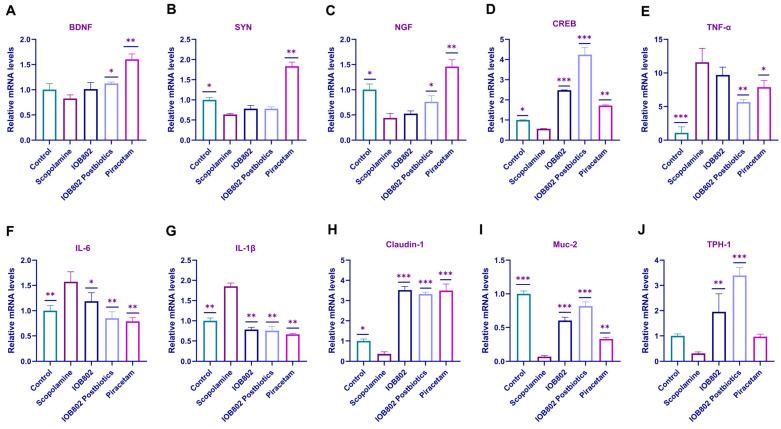
The expression of relevant factors in the hippocampus and colon of mice. (**A**) The expression of BDNF in hippocampal tissue. (**B**) The expression of SYN in hippocampal tissue. (**C**) The expression of NGF in hippocampal tissue. (**D**) The expression of CREB in hippocampal tissue. (**E**) The expression of TNF-α in colonic tissue. (**F**) The expression of IL-6 expression in colonic tissue. (**G**) The expression of IL-1β in colonic tissue. (**H**) The expression of Claudin-1 in colonic tissue. (**I**) The expression of Muc-2 in colonic tissue. (**J**) The expression of TPH-1 in colonic tissue. Data represent mean ± SD. Statistical significance was assessed using one-way ANOVA followed by Dunnett’s post hoc test. Compared to the Scopolamine group, * *p* < 0.05, ** *p* < 0.01, and *** *p* < 0.001.

Neurotrophic factors and inflammation play important roles in cognitive function. In Alzheimer’s and Parkinson’s diseases, the levels of brain-derived neurotrophic factor (BDNF), synaptophysin (SYN), and nerve growth factor (NGF) are reduced, leading to impaired synaptic plasticity as well as decreased neuronal density and cholinergic neuron survival in the hippocampus [38]. Moreover, systemic inflammation triggered by increased gut permeability and gut microbiota dysbiosis may accelerate neurodegenerative processes. Given that CREB is a key upstream transcriptional regulator of BDNF and a crucial mediator of memory consolidation, we also examined the effect of IOB802 on CREB expression. Assessment of relevant markers in both the hippocampus and colon revealed that scopolamine treatment markedly decreased hippocampal levels of BDNF, SYN, NGF, and CREB (Figure 5A–D), while simultaneously increasing colonic expression of pro-inflammatory cytokines TNF-α, IL-6, and IL-1β (Figure 5E–G). To further evaluate gut permeability associated with systemic inflammation, we analyzed tight junction proteins and mucosal barrier integrity. The results showed that scopolamine significantly reduced colonic mRNA expression of a tight junction component Claudin-1 and intestinal mucin MUC-2, indicating impaired intestinal barrier function (Figure 5H,I). In addition, scopolamine decreased the expression of tryptophan hydroxylase 1 (TPH-1) in the colon, suggesting disrupted gut serotonin synthesis (Figure 5J). Treatment with IOB802 and its postbiotics significantly upregulated Claudin-1, MUC-2, and TPH-1 mRNA levels, restored hippocampal expression of neurotrophic factors and CREB, and suppressed pro-inflammatory cytokines. Additionally, we measured the protein expression levels of memory-related markers BDNF and p-CREB using ELISA. The findings indicated that scopolamine significantly reduced the protein levels of BDNF and p-CREB in the hippocampus, whereas IOB802 and its postbiotic notably enhanced the protein levels of BDNF and p-CREB (Figure S3). These findings suggest that IOB802 and its postbiotics can simultaneously enhance central nervous system plasticity, improve intestinal barrier integrity, and modulate gut serotonin metabolism.

### 3.7. IOB802 and Its Postbiotics Affected Gut Microbial Diversity in Mice

To assess the impact of *Limosilactobacillus fermentum* IOB802 and its postbiotics on the gut microbiota of cognitively impaired mice, the V3–V4 region of the 16S rRNA gene in colonic content samples was sequenced. Then alpha diversity metrics like the Ace index, Chao1 index, and Shannon index were used to assess the microbiota’s abundance and diversity. As illustrated in Table 3, the coverage values were higher than 0.999 for all groups, indicating that the sequencing depth was enough to capture adequate bacterial community. The Ace and Chao1 metrics indicated a decrease in microbiome richness due to scopolamine, whereas IOB802 Postbiotics and Piracetam partially or completely restored the microbiome richness. The community diversity indicated by the Shannon indexes showed a slight decrease in microbial diversity due to scopolamine, when an increase in microbial diversity was observed after IOB802 postbiotics and Piracetam treatment. However, Simpson index showed no significant changes, except that Piracetam caused a decrease, among the different groups (Table 3). These results suggested that IOB802 postbiotics affected gut microbial richness and diversity.

### 3.8. IOB802 and Its Postbiotics Have an Effect on the Gut Microbial Composition

**Figure 6:**
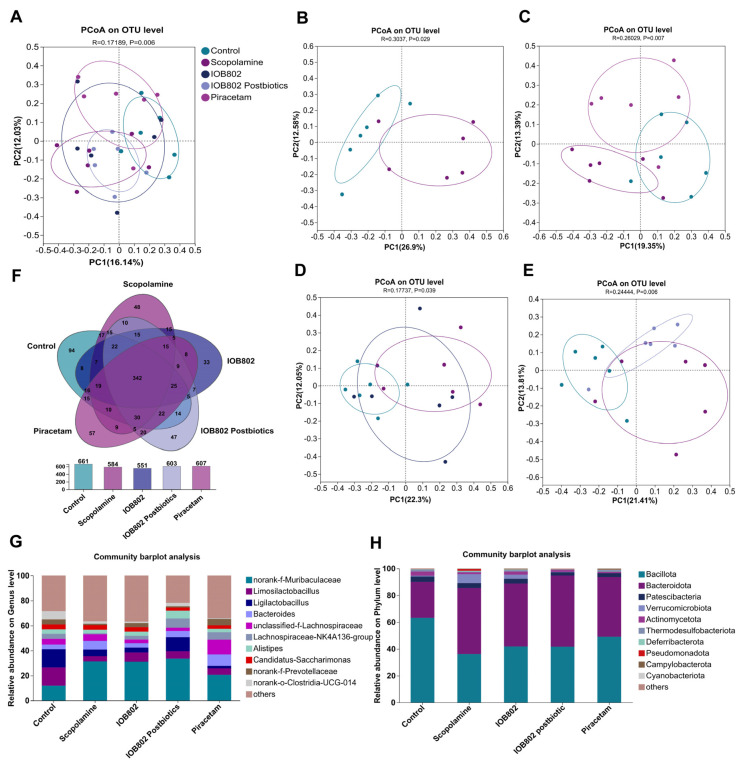
Functional and structural modulation of the gut microbiota by *Lacticaseibacillus mucosae* IOB802 in a mouse model of cognitive impairment. (**A**,**B**) Principal coordinate analysis (PCoA) based on Bray–Curtis distance at the OTU level for all mice and for Control vs. Scopolamine groups. (**C**–**E**) PCoA (Bray–Curtis, OTU level) among Control, Scopolamine, IOB802, IOB802-postbiotics and Piracetam groups. (**F**) Venn diagram of species-level microbial composition across groups. (**G**) Genus-level microbiota composition among groups. (**H**) Phylum-level microbiota composition among groups. Data represent mean ± SD (n = 6/group). Statistical significance was assessed using one-way ANOVA followed by Dunnett’s post hoc test. Compared to the Scopolamine group.

Gut dysbiosis has been increasingly recognized as a pathological feature associated with cognitive impairment, where reduced microbial diversity and abnormal shifts in specific taxa contribute to neuroinflammation, altered neurotransmitter metabolism, and impaired synaptic plasticity through the microbiota–gut–brain axis [39]. To assess the impact of IOB802 and its postbiotic on microbial community structure, we conducted a comparative analysis. Principal coordinate analysis (PCoA) revealed a distinct separation between the scopolamine-treated and control groups, confirming that scopolamine administration induced gut microbial dysbiosis. Notably, the IOB802 postbiotic group showed partial overlap with, yet remained distinguishable from, the scopolamine group, indicating that postbiotic intervention led to a partial recovery of the gut microbial community (Figure 6A–E). At the OTU level, a total of 342 OTUs were shared among the five groups, while each group also exhibited unique OTUs, suggesting the presence of both conserved and group-specific taxa (Figure 6F).

At the phylum level, *Bacillota*, *Bacteroidota*, *Verrucomicrobiota*, and *Actinomycetota* represented the predominant taxa across all groups (Figure 6H). Compared to the Control group, scopolamine treatment significantly reduced the abundance of *Bacillota* and *Actinomycetota*, while it increased the abundance of *Bacteroidota* and *Verrucomicrobiota*. As *Bacillota* are major producers of short-chain fatty acids, their reduction may compromise host energy metabolism and immune regulation [40]. In contrast, elevated *Verrucomicrobiota* abundance has frequently been associated with pro-inflammatory states in experimental models [41]. Administration of IOB802 and its postbiotics mitigated these alterations, characterized by suppression of *Verrucomicrobiota* and recovery of *Actinomycetota*, indicating a capacity to restore microbial balance and alleviate inflammation. At the genus level, scopolamine administration increased the relative abundances of *Bacteroides* and unclassified *Lachnospiraceae*, while markedly reducing the probiotic genera *Limosilactobacillus*, *Ligilactobacillus*, *Lachnospiraceae_NK4A136_group*, and *Alistipes* (Figure 6G). In addition, scopolamine treatment also altered several other genera, characterized by increased abundances of *Butyricicoccus*, *Mediterraneibacter*, *Enterococcus*, and *unclassified Prevotellaceae*, accompanied by reductions in *Akkermansia*, *Lactobacillus*, *Roseburia*, and *Odoribacter*, indicating a more widespread disturbance of the gut microbial community (Figure S4). An excessive representation of *Bacteroides* has previously been linked to elevated pro-inflammatory cytokines in models of cognitive dysfunction, whereas *Alistipes* and *lactobacilli* are generally associated with anti-inflammatory effects and maintenance of metabolic homeostasis [42,43]. Treatment with IOB802 and its postbiotics counteracted these scopolamine-induced changes by suppressing *Bacteroides* and unclassified *Lachnospiraceae*, while restoring *Limosilactobacillus*, *Ligilactobacillus*, and *Alistipes*. These findings suggest that IOB802 interventions promote the re-establishment of microbial balance and the recovery of health-associated taxa.

### 3.9. IOB802 and Its Postbiotics Affect Short-Chain Fatty Acids (SCFAs) Metabolic Profiling

**Figure 7:**
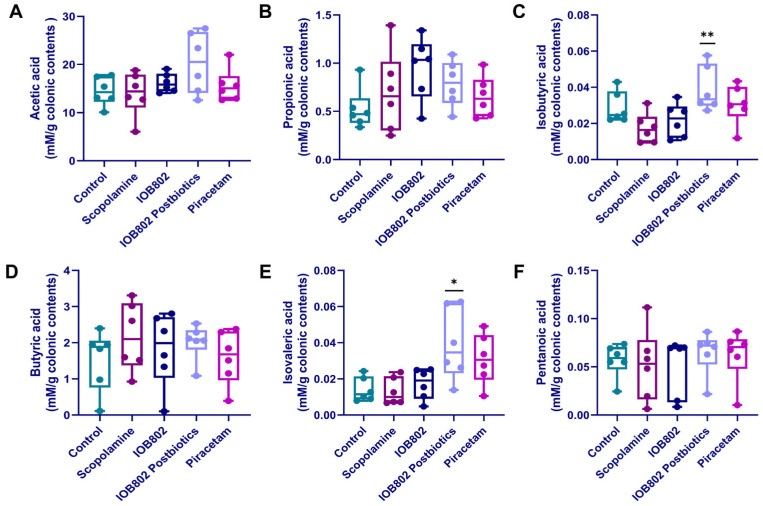
Fecal short-chain fatty acid profile in cognitively impaired mice after IOB802 or IOB802 postbiotic intervention. (**A**) Acetic acid; (**B**) Propionic acid; (**C**) Isobutyric acid; (**D**) Butyric acid; (**E**) Isovaleric acid; (**F**) Pentanoic acid. Data represent mean ± SD (n = 6/group). Statistical significance was assessed using one-way ANOVA followed by Dunnett’s post hoc test. Compared to the Scopolamine group, * *p* < 0.05, ** *p* < 0.01.

Emerging evidence indicates that gut-derived short-chain fatty acids (SCFAs) and branched-chain fatty acids (BCFAs) modulate neuroinflammation and synaptic plasticity through the gut–brain axis, thereby influencing cognitive performance [44]. To investigate the effects of IOB802 and its postbiotics on SCFA metabolic profiling, the cecum samples of scopolamine-induced cognitive-impairment model were analyzed quantitatively for the six SCFAs. Total SCFA levels slightly decreased in the scopolamine group compared to the control, while significantly increased in the IOB802 postbiotics group compared to the scopolamine group (Figure S5). The concentration of acetate, propionate, butyrate and valerate did not differ among groups, indicating that scopolamine did not affect the biosynthesis of these major SCFAs (Figure 7A,B,D,F). In contrast, IOB802 postbiotics selectively enhanced the production of BCFA: isobutyrate (Figure 7C) and isovalerate (Figure 7E) were significantly elevated relative to the scopolamine group, whereas the parental IOB802 strain left the fecal fatty acid profile unchanged. Collectively, these data reveal that, although scopolamine does not markedly disturb principal SCFA levels, IOB802 postbiotics specifically promote BCFA formation—an effect that may reshape the intestinal milieu and attenuate neuroinflammation, thereby offering a plausible metabolic mechanism underpinning the observed cognitive benefit.

## 4. Discussion

Cognitive impairment, serving as a transitional phase between normal aging and AD, presents with memory deficits, other cognitive impairments, and alterations in emotions and personality. Despite being mild and having limited impact on daily functioning, these symptoms pose challenges in detection and diagnosis. Studies have reported that approximately 10–20% of patients with cognitive impairment transition to AD each year [45]. Currently, there is still a lack of effective treatments for AD and cognitive impairment. Therefore, preventing the occurrence of cognitive impairment can serve as an effective entry point to directly reduce the incidence of AD [46]. Growing evidence positions the gut–brain axis as a key driver in the onset and progression of central nervous system disorders. Gut microbes modulate neurotransmitter availability, immune–inflammatory tone and synaptic plasticity through bioactive metabolites, thereby shaping cognitive performance [47]. Here, using a scopolamine-induced mouse model of cognitive impairment, we systematically evaluated the cognitive benefits and underlying mechanisms of *Limosilactobacillus fermentum* IOB802—originally isolated from kimchi—and its postbiotics.

Cognitive decline is intimately linked to cholinergic hypofunction and neurotransmitter dysregulation: diminished central acetylcholine (ACh) synthesis coupled with hyper-activated acetylcholinesterase (AChE) erodes cholinergic synaptic transmission, while the homeostatic balance between excitatory and inhibitory signals (glutamate vs. GABA) and monoaminergic tone (5-HT, DA, NE) is disrupted [48]. The resulting excitation–inhibition imbalance impairs synaptic plasticity and neuronal synchronization, triggering oxidative stress, mitochondrial failure and a neuroinflammatory cascade that ultimately drives apoptosis and synaptic loss in the hippocampus and prefrontal cortex, manifesting as multi-domain cognitive deficits [49]. Here, IOB802 and its postbiotics markedly reversed scopolamine-induced learning and memory impairments: treated mice exhibited shorter escape latencies in the Morris water maze and fewer errors in the step-down test, reflecting restored spatial and passive-avoidance memory. Neurochemical analyses revealed elevated cortico-hippocampal ACh and suppressed AChE activity, indicating rescued cholinergic signaling. Concomitantly, GABA and 5-HT levels were re-balanced, supporting the reinstatement of transmitter homeostasis. However, the differential effects of IOB802 versus its postbiotic on serum 5-HT levels were discovered in this study. The postbiotic showed superior efficacy in restoring 5-HT, and the effect may be influenced by the following mechanisms including bioactive metabolite enrichment, gut transit considerations, host immune modulation and tryptophan metabolism [50]. Importantly, serum transaminases remained unchanged, underscoring the safety of the intervention. Collectively, IOB802 and its postbiotics improve cognition by re-normalizing cholinergic function and neurotransmitter equilibrium, providing new experimental evidence for probiotic-based strategies against cognitive dysfunction.

Oxidative stress is a recognized driver of cognitive decline, in which superoxide dismutase (SOD) and malondialdehyde (MDA) serve as key biomarkers. Depleted SOD and concomitant elevation of MDA reflect compromised antioxidant defenses and excess free-radical accumulation, events that provoke neuronal injury and synaptic dysfunction [51]. Additionally, the accumulation of MDA may harm neuronal cell membranes, leading to decreased membrane fluidity and impaired organelle function. These effects can disrupt normal neuronal function and information transmission, ultimately exacerbating cognitive impairment [52]. Alejandra Romo-Araiza et al. utilized *Lactobacillus rhamnosus* as a supplement to curcumin, observing its ability to mitigate abnormal SOD levels in brain tissue induced by scopolamine [45]. Hamideh Rahmati et al. administered a capsule comprising seven mixed probiotics to investigate the therapeutic impact of probiotics on MDA abnormalities in a murine model of cerebral hypoperfusion [7]. In the present study, scopolamine decreased cortico-hippocampal SOD activity while raising MDA levels, whereas IOB802 and its postbiotics fully reversed these anomalies, restoring endogenous antioxidant capacity and curbing lipid peroxidation. Taken together, IOB802 and its postbiotics counteract oxidative stress-mediated neurodamage by reinstating SOD activity and suppressing MDA accumulation, offering a novel antioxidant mechanism through which probiotics may alleviate cognitive impairment.

Histological analyses corroborated these findings. The hippocampus, situated in the innermost region of the temporal lobe, is a crucial component of the human memory system within the limbic system [53]. Damage to the hippocampus can lead to emotional disturbances and memory deterioration. Cognitive impairments such as vascular dementia (VaD) [54] or obstructive sleep apnea syndrome (OSAS) [55] can result in hippocampal neuronal cell injury, characterized by nuclear pycnosis, disorganized cell arrangement, indistinct boundaries, widened intercellular spaces, irregular cell morphology, and an increase in neurodegenerative and necrotic cells [56]. In the present study, scopolamine induced overt neuronal malformation, whereas IOB802 and its postbiotics markedly attenuated these pathological changes, restoring structural integrity and cellular viability. Moreover, elevated neurotrophic factors in brain tissue and diminished pro-inflammatory cytokines in colon tissue demonstrated that IOB802 and its postbiotics simultaneously enhance neuroprotection and suppress peripheral inflammation. Thus, beyond ameliorating neuronal morphology, IOB802 mitigates global inflammatory burden via peripheral immune modulation during cognitive impairment.

Disruption of the gut microbiota compromises the intestinal barrier and reduces the production of SCFAs. SCFAs serve as essential energy substrates for intestinal epithelial cells and can cross the blood–brain barrier. By activating FFAR2/3 receptors and inhibiting the NF-κB signaling pathway, SCFAs mitigate neuroinflammation and support synaptic plasticity, which are beneficial for cognitive performance [57]. Therefore, alterations in gut microbial composition and SCFA levels following probiotic intervention are often regarded as key indicators for assessing the involvement of the microbiota–gut–brain axis in cognitive regulation. The integrity of the intestinal barrier serves as a critical link within this axis. Dysbiosis not only affects microbial metabolism but can also impair the mucus layer and reduce the expression of tight junction proteins, such as Muc-2 and members of the claudin family, resulting in increased epithelial permeability [58]. Loss of barrier integrity permits pathogen-associated molecular patterns from the gut lumen to enter the circulation more readily, eliciting systemic inflammation and potentially driving peripheral inflammatory signals toward the central nervous system [59]. This cascade may worsen neuroinflammation and contribute to cognitive deterioration. Consequently, the status of the intestinal barrier represents a key biological node connecting microbial disturbances with downstream neuropathological outcomes [60]. In this study, IOB802 postbiotics markedly influenced the gut microbial community and intestinal barrier. Treatment with IOB802 postbiotics improved microbial composition by enhancing the growth of beneficial taxa and restricting potential pathogens. Moreover, IOB802 and IOB802 postbiotics enhancing the expression of Claudin-1 and MUC-2, which were the key genes for the intestinal barrier. Metabolite profiling further showed an increase in branched-chain fatty acids (BCFAs) following treatment. These results indicate that IOB802 may contribute to remodeling the gut environment, intestinal barrier and altering metabolite production, which could secondarily affect central nervous system function.

To conclude, this study explored the potential mechanisms by which IOB802 and its postbiotics may alleviate cognitive impairment. The effects of IOB802 were associated not only with the restoration of cholinergic system homeostasis, enhanced antioxidant defense, and suppression of inflammatory responses, but also with improvements in gut microbiota composition and increased short-chain fatty acid production. The interplay of these mechanisms may help preserve neuronal integrity, improve synaptic function, and support cognitive recovery. These findings provide experimental evidence for the application of IOB802 as a functional probiotic in cognitive impairment and point to new research directions for microbiota–gut–brain axis–based strategies in cognitive regulation.

## 5. Conclusions

**Figure 8:**
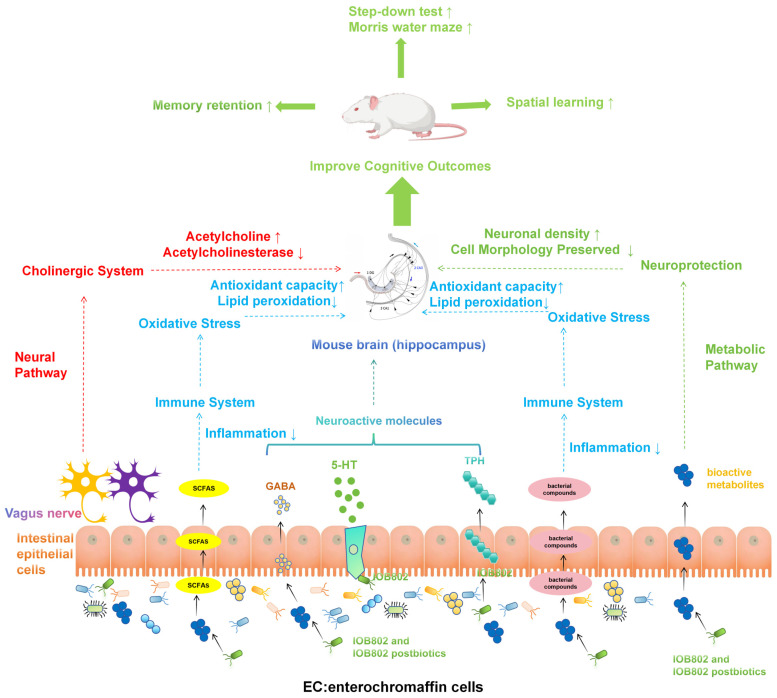
Mechanism diagram of the improvement effect of IOB802 and IOB802 postbiotic on cognitive impairment in mice through the brain–gut axis.

In summary, IOB802 has been identified as a promising probiotic strain for cognitive intervention. IOB802 acts through the gut–brain axis to enhance cholinergic transmission, increase ACh levels, and inhibit AChE activity. Additionally, it elevates GABA and 5-HT levels to restore neurotransmitter balance and reduce excitatory–inhibitory dysregulation. These changes help protect against oxidative stress and lipid peroxidation damage, leading to alleviation of neuronal degeneration in the hippocampus. Furthermore, IOB802 reduces inflammatory mediators and activates neuroprotective factors to suppress neuroinflammation. IOB802 and its postbiotics enhance beneficial bacteria, suppress harmful microbes, and increase the production of bioactive compounds like branched-chain fatty acids (Figure 8). These changes indirectly support brain function and immune system balance, leading to improved cognitive performance. Overall, IOB802 and its postbiotics regulate the gut–brain axis in a coordinated manner, providing evidence for their use as a probiotic for cognitive issues. This highlights the potential of targeting the microbiota-gut–brain axis for cognitive enhancement strategies.

## Figures and Tables

**Table 1:** Primer Sequences.

GenBank Accession Number	Primer	Sequences (5′→3′)	Amplicon Size
NM_001289726.2	GAPDH	Forward: GGCTGTATTCCCCTCCATCG	154
		Reverse: CCAGTTGGTAACAATGCCATGT	
NM_001278601.1	TNF-α	Forward: GACGTGGAACTGGCAGAAGAG	228
		Reverse: TTGGTGGTTTGTGAGTGTGAG	
NM_001314054.1	IL-6	Forward: CCAAGAGGTGAGTGCTTCCC	118
		Reverse: CTGTTGTTCAGACTCTCTCCCT	
NM_008361.4	IL-1β	Forward: GCAACTGTTCCTGAACTCAACT	89
		Reverse: ATCTTTTGGGGTCCGTCAACT	
NM_001048139.1	BDNF	Forward: TCATACTTCGGTTGCATGAAGG	137
		Reverse: AGACCTCTCGAACCTGCCC	
NM_009305.2	SYN	Forward: CAGTTCCGGGTGGTCAAGG	138
		Reverse: ACTCTCCGTCTTGTTGGCAC	
NM_001112698.2	NGF	Forward: CCAGTGAAATTAGGCTCCCTG	142
		Reverse: CCTTGGCAAAACCTTTATTGGG	
NM_001037726.1	CREB	Forward: CAGGGGTCGCAAGGATTGAAG	125
		Reverse: ATCGCCTGAGGCAGTGTACT	
NM_001136084.2	TPH1	Forward: AACAAAGACCATTCCTCCGAAAG	119
		Reverse: TGTAACAGGCTCACATGATTCTC	
NM_016674.4	Claudin-1	Reverse: CCGGATAAAAAGAGTACGCTGG	100
		Forward: GGGGACAACATCGTGACCG	
NM_023566.4	Muc-2	Reverse: AGGAGTCGAAGACTTTGCACT	106
		Forward: AGGGCTCGGAACTCCAGAAA	

**Table 2:** Incubation period and the number of errors in Step-Down test.

Group	Incubation Period (s)	Number of Errors
Control	114.61 ± 4.62 *	0.71 ± 0.49 *
Scopolamine	81.52 ± 21.99	2.40 ± 1.14
IOB802	105.96 ± 12.20 *	0.88 ± 0.35 *
IOB802 postbiotic	108.39 ± 9.80 *	1.00 ± 0.63 *
Piracetam	105.27 ± 11.36 *	0.90 ± 0.32 *

Mean ± SD data from 6 mice in each group were presented. * *p* < 0.05, compared to the Scopolamine group. Statistical significance was assessed using one-way ANOVA, followed by Dunnett’s post hoc test.

**Table 3:** IOB802 and IOB802 postbiotics affected alpha diversity indices in mice.

Group	Chao 1	Ace	Simpson	Shannon	Coverage
Control	324.12 ± 48.16	325.81 ± 52.12	0.13 ± 0.12	3.28 ± 0.75	0.9993
Scopolamine	305.68 ± 52.27	307.92 ± 53.05	0.10 ± 0.05	3.24 ± 0.32	0.9992
IOB802	281.04 ± 47.52	285.82 ± 51.70	0.11 ± 0.06	3.15 ± 0.53	0.9993
IOB802 Postbiotics	362.67 ± 44.24	362.69 ± 47.73	0.10 ± 0.09	3.53 ± 0.62	0.9993
Piracetam	317.42 ± 42.93	321.37 ± 41.77	0.07 ± 0.03	3.51 ± 0.29	0.9992

Mean ± SD data from 6 mice in each group were presented.

## Data Availability

The original contributions presented in this study are included in the article/Supplementary Materials. Further inquiries can be directed to the corresponding authors.

## Supplementary Materials

The following supporting information can be downloaded at https://www.mdpi.com/article/10.3390/foods14234037/s1, Table S1: The antimicrobial-susceptibility profiles of IOB802; Figure S1: Schematic illustration of the treatment of KM mice; Figure S2: Changes in body weight over time in different treatment groups; Figure S3: The protein expression levels of memory-associated markers BDNF and p-CREB in the hippocampus of mice; Figure S4: Heatmap analysis of gut microbiota composition at the genus level across treatment groups; Figure S5: Concentrations of short-chain fatty acids (SCFAs) in the colon of different treatment groups.
