# Supplementary material for: A Novel Probiotic Limosilactobacillus fermentum IOB802 and Its Postbiotic Alleviate Cognitive Impairment Induced by Scopolamine in Mice
Source: Foods. 2025 Nov 25;14(23):4037. doi: 10.3390/foods14234037 (PMC12692042; doi:10.3390/foods14234037)
Supplement: Supplementary file 1 [file foods-14-04037-s001.zip › foods-3967044-supplementary.pdf]

Table S1. The antimicrobial-susceptibility profiles of IOB802

Antibiotic designations	Disk content (μg per disk)	Zone-diameter interpretative criteria (mm)			IOB802	
		R (Resistance)	I (Intermediary)	S (Sensitive)	Zone-diameter (mm)	Result
Ampicillin (AMP)	10	≤13	14-16	≥17	22.23±1.70	S
Clarithromycin (CLR)	15	≤13	14-17	≥18	19.35±3.44	S
Oxacillin (OX)	1	≤10	11-12	≥13	12.85±0.39	I
Clindamycin (CC)	2	≤15	16-18	≥19	15.24±1.57	R
Fleroxacin (FLE)	5	≤15	16-18	≥19	-	R
Erythromycin (E)	15	≤13	14-22	≥23	18.81±1.66	I
Streptomycin (S)	10	≤11	12-14	≥15	-	R

Data are presented as Mean ± SD (n = 3 per group). Zone-diameter interpretative criteria refer to the measurement of the transparent inhibition zone diameter (mm) around an antibiotic disk.

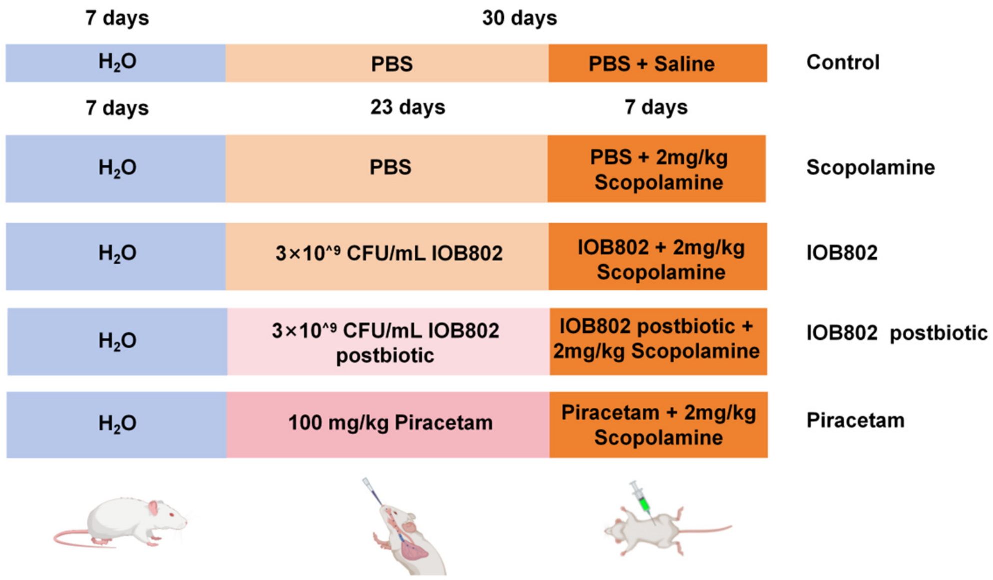

Figure S1. Schematic illustration of the treatment of KM mice.

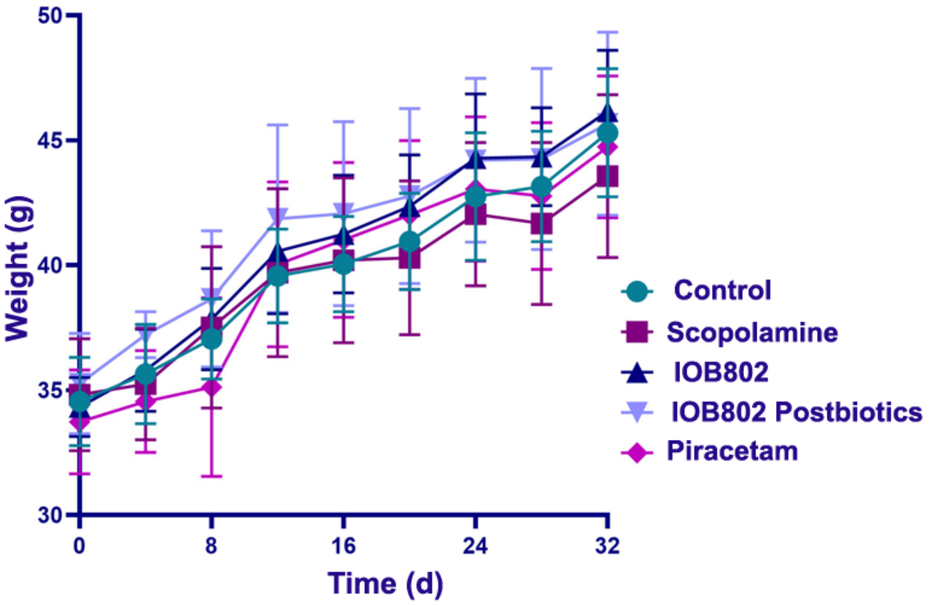

Figure S2. Changes in body weight over time in different treatment groups of mice.

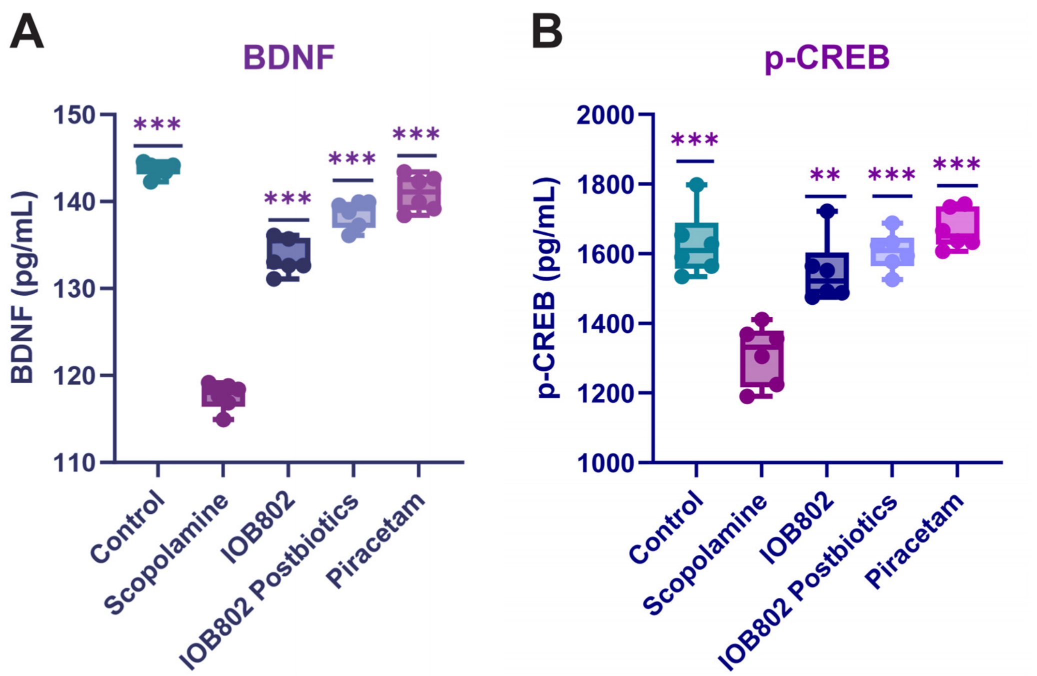

Figure S3. The protein expression levels of memory-associated markers BDNF and p-CREB in the hippocampus of mice.(A) The protein level of BDNF in brain tissue. (B) The p-CREB level in brain tissue. Data represent mean ± SD (n = 6 per group). Statistical significance was assessed using one-way ANOVA followed by Dunnett's post-hoc test. Compared to the Scopolamine group, ** $p < 0.01$ and *** $p < 0.001$.

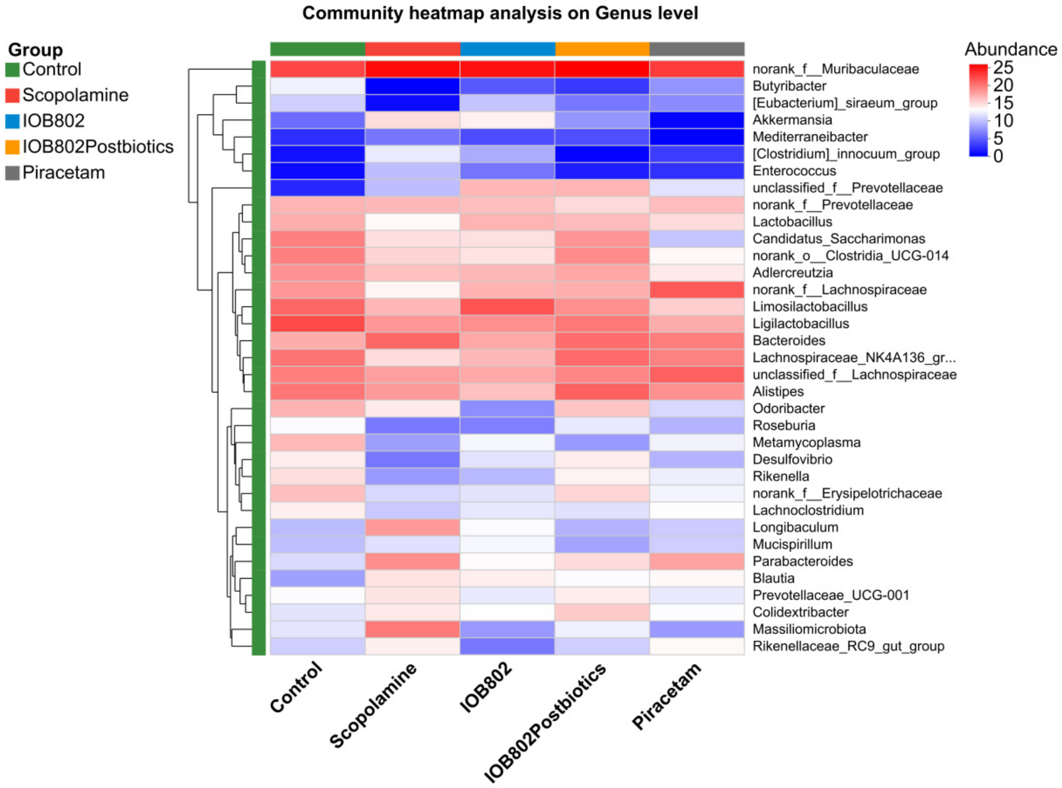

Figure S4. Heatmap analysis of gut microbiota composition at the genus level across treatment groups of mice.

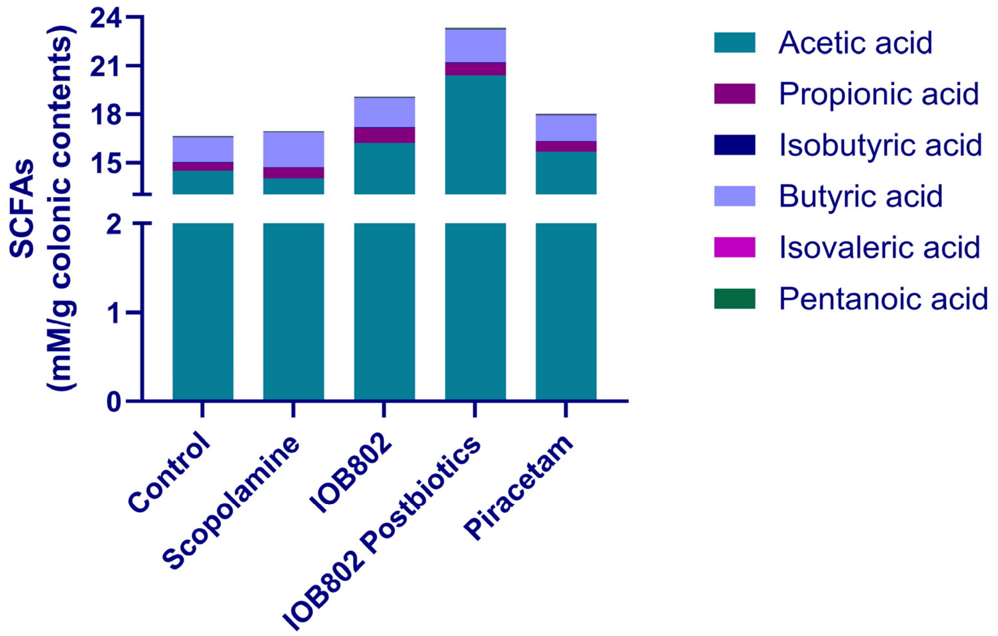

Figure S5. Concentrations of short-chain fatty acids (SCFAs) in the colon of different treatment groups of mice.
